# The Extracellular Vesicles Proteome of Endometrial Cells Simulating the Receptive Menstrual Phase Differs from That of Endometrial Cells Simulating the Non-Receptive Menstrual Phase

**DOI:** 10.3390/biom13020279

**Published:** 2023-02-02

**Authors:** Amber Rose Hart, Norhayati Liaqat Ali Khan, Keerthie Dissanayake, Kasun Godakumara, Aneta Andronowska, Saji Eapen, Paul R Heath, Alireza Fazeli

**Affiliations:** 1 Academic Unit of Reproductive and Developmental Medicine, Department of Oncology and Metabolism, The Medical School, University of Sheffield, Jessop Wing, Tree Root Walk, Sheffield S10 2SF, UK; 2 Faculty of Dentistry, University Teknologi MARA (UiTM), Shah Alam 40450, Malaysia; 3 Department of Pathophysiology, Institute of Biomedicine and Translational Medicine, Faculty of Medicine, University of Tartu, Ravila St. 14B, 50411 Tartu, Estonia; 4 Institute of Veterinary Medicine and Animal Sciences, Estonian University of Life Sciences, Kreutzwaldi 62, 51006 Tartu, Estonia; 5 Department of Anatomy, Faculty of Medicine, University of Peradeniva, Peradeniva 20400, Sri Lanka; 6 Institute of Animal Reproduction and Food Research, Polish Academy of Sciences, Juliana Tuwima St. 10, 10-748 Olsztyn, Poland; 7 SPD Development Co., Ltd., Clearblue Innovation Centre, Stannard Way, Priory Business Park, Bedford MK44 3UP, UK; 8 Department of Neuroscience, University of Sheffield, Sheffield S10 2HQ, UK

**Keywords:** extracellular vesicles, endometrium, receptivity, fertility, proteomics

## Abstract

Successful embryo implantation into a receptive endometrium requires mutual endometrial-embryo communication. Recently, the function of extracellular vehicles (EVs) in cell-to-cell interaction in embryo-maternal interactions has been investigated. We explored isolated endometrial-derived EVs, using RL95-2 cells as a model of a receptive endometrium, influenced by the menstrual cycle hormones estrogen (E2; proliferative phase), progesterone (P4; secretory phase), and estrogen plus progesterone (E2P4; the receptive phase). EV sized particles were isolated by differential centrifugation and size exclusion chromatography. Nanoparticle tracking analysis was used to examine the different concentrations and sizes of particles and EV proteomic analysis was performed using shotgun label-free mass spectrometry. Our results showed that although endometrial derived EVs were secreted in numbers independent of hormonal stimulation, EV sizes were statistically modified by it. Proteomics analysis showed that hormone treatment changes affect the endometrial EV’s proteome, with proteins enhanced within the EV E2P4 group shown to be involved in different processes, such as embryo implantation, endometrial receptivity, and embryo development, supporting the concept of a communication system between the embryo and the maternal endometrium via EVs.

## 1. Introduction

The development of the mammalian embryo into a complete organism is dependent on the successful implantation of the embryo into the uterus wall within a limited window of implantation during the menstrual cycle. In humans, the menstrual cycle refers to cyclic changes under the control of several molecules, including the sex hormones, estrogen and progesterone, working in a negative and positive feedback mechanism [1]. These resulting changes in the endometrium lead to a receptive period during the secretory phase for blastocyst arrival and implantation. However, there is only around a 30% chance of conception occurring in one menstrual cycle; such a low percentage is due to many factors including the short receptive period of 4–5 days [2]. Studies have suggested that by controlling these cyclic changes, the endometrium modulates the uterus environment leading to the secretion of various molecules, factors, and proteins that aid in conception [3]. Accordingly, this complex process of receptive endometrial remodelling and embryo implantation likely requires a sophisticated exchange of signals between embryo and endometrium [4]. However, the mechanisms involved in this process are not yet fully understood.

Recently, extracellular vesicles (EVs), which are lipid bilayer particles secreted into the extracellular space, have been shown to be released from the endometrium and to have a key functional role in cell-to-cell communication, including embryo-maternal interactions [5,6,7,8]. The cargos and size distribution of endometrial EVs at different phases of the cycle could affect the functionality of EVs. In terms of the physical properties of vesicles, including size and distribution, these factors previously have been shown to have a determining effect on the recipient cell ability to be internalised [9]. Regarding cargo, endometrial EVs are known to contain various biological molecules, such as proteins, lipids, and nucleic acids, including mRNA, with studies beginning to suggest that they may be up taken by trophoblastic cells, with this content being transferred to the blastocyst to improve or aid in the invasion or adhesion capacity of the embryo during the implantation process [10,11,12]. The cargos of endometrial EVs, including proteins, DNA, and RNA, have previously been shown in various models [13,14,15]; however, throughout the menstrual cycle phase of humans, the question remains if the proteomic profile and the size of the endometrial EVs differ or remains constant. This recent experimental data emphasises the role that endometrial signalling may play via EVs on the preparation of the receptive endometrium for embryo implantation. The current investigation builds on this data and aims to identify the characteristics of endometrial EVs at different phases of the menstrual cycle by testing the hypothesis that the characteristics of endometrial EVs change across the different stages of the menstrual cycle. This information has huge potential in clinical applications in therapeutics and biomarker discovery for assisted reproductive treatments.

Many studies have employed in vitro cell culture systems to advance the understanding of the role of EVs in the female reproductive tract and the intercellular communications [8,16,17]. To produce beneficial conclusions from such experiments, it is paramount that cell culture models are optimised for the production and recovery of EVs, whilst mimicking the true in vivo conditions as much as possible. To clarify the mechanisms involved in implantation success and endometrial receptivity, in the present study we isolated EVs secreted by RL95-2 cells under conditions that mimic the menstrual cycle phases and analysed their protein content via proteomic approaches. This study revealed new characteristics and dynamics of endometrial EVs at different phases of the menstrual cycle and thus revealed physiological and pathological changes associated with the menstrual cycle.

## 2. Materials and Methods

### 2.1. Cell Culture

Two types of media have been used: (i) Dulbecco’s Modified Eagles Medium (DMEM/F12) culture media (Sigma-Aldrich, Dorset, UK), supplemented with 10% fetal bovine serum (FBS) (Sigma-Aldrich, Dorset, UK), 1% l-glutamine (Sigma-Aldrich, Dorset, UK), 1% penicillin (100 IU/mL)/streptomycin (100 µg/mL) (Sigma-Aldrich, Dorset, UK), and 5 µg/mL insulin (human recombinant insulin; Gibco Invitrogen, Roskilde, Denmark); and (ii) Dulbecco’s Modified Eagles Medium lacking phenol red (Thermo Fisher, Scotland, UK). The final variation (iii) was phenol red free media (ii) with charcoal stripped FBS (10%) (Sigma-Aldrich, Dorset, UK).

RL95-2 cells obtained from American Type Culture Collection (ATCC, Cat. No. CRL-1671, Teddington, UK) were used to simulate receptive endometrial cells in vitro. RL95-2 cells were cultured in T75 flasks (Greiner, bio-one, Gloucestershire, UK) in culture media (i). Cells were incubated at 37 °C in a 5% CO_2_ atmosphere until confluent. At confluence, cells were washed using Ca^2+^ and Mg^2+^ free Dulbecco’s phosphate-buffered saline (Sigma-Aldrich, Dorset, UK). The cells were then harvested using trypsin-EDTA (Sigma-Aldrich, Dorset, UK) and pelleted by centrifugation at 300× *g* for 4 min.

### 2.2. Differential Centrifugation

Conditioned media was centrifuged for 10 min at 400× *g* at 4 °C in a Sigma swinging-out rotor centrifuge (catalogue number 11,180). The pellet was discarded, and the supernatant was centrifuged for 10 min at 4000× *g* at 4 °C; again the pellet was discarded, and the supernatant was centrifuged for 10 min at 10,000× *g* at 4 °C. The supernatant was then stored at −80 °C for further isolation procedures and experiments. An aliquot was reserved from the control group as a conditioned media control.

### 2.3. Size Exclusion Chromatography

For the preparation of a size exclusion chromatography (SEC) column, 14-mL of Sepharose CL-2B slurry (GE Healthcare, Uppsala, Sweden) was poured into an empty gravity chromatography column (Bio-Rad, Watford, UK). After settling of the Sepharose beads, a nylon net filter was inserted at the ethanol/Sepharose separation interface at about 10 mL. The ethanol was eluted followed by washing the column with molecular grade water (3 × 5 mL) and then with Ca^2+^ and Mg^2+^ free Dulbecco’s phosphate-buffered saline (3 × 5 mL) (Sigma-Aldrich, Dorset, UK). On top of the filter, 0.5 mL of the concentrated conditioned medium was loaded, and fractions of 0.5 mL were collected in 20 fractions with a Ca^2+^ and Mg^2+^ free Dulbecco’s phosphate-buffered saline eluent. The resulting fractions were stored at −80 °C for further EV characterisation.

### 2.4. Nanoparticle Tracking Analysis

All samples were diluted in molecular grade water. Ideal measurement concentrations were found by pre-testing the ideal particles per frame value (140–200 particles/frame). For each measurement, three cycles were performed by scanning 11 cell positions each and capturing 30 frames per position under the following settings: focus: autofocus; camera sensitivity for all samples: 50; shutter: 70; scattering intensity: detected automatically; and cell temperature: 25 °C. After capture, the videos were analysed by the in-built ZetaView Software 8.04.02 SP2 with specific analysis parameters: maximum size: 500; minimum size: 5; minimum brightness: 25; hardware: embedded laser: 40 mW at 488 nm; and camera: CMOS.

### 2.5. RL95-2 Cell Conditioned Media EV Isolation Optimisation

This experiment was carried out to determine which of the size exclusion fractions contain EV particles. RL95-2 cells were cultured in T75 flasks with complete DMEM culture media until ~70/80% confluent. Cells were washed using DPBS without Ca^2+^ and Mg^2+^ 3 times and FBS free Dulbecco’s Modified Eagles Medium (DMEM/F12) culture media added and incubated for 24 h. After 24 h the conditioned media was collected, and differential centrifugation was performed. Conditioned media underwent differential centrifugation followed by concentration using a 10 kDa ultrafiltration tube (Sartorius, Hampshire, UK) to 500 µL. Retentates were subjected to size exclusion chromatography with 20 × 0.5 mL fractions collected. The protein content of the fractions was determined using the bicinchoninic acid assay kit (Pierce™ BCA Protein Assay Kit, Thermo Scientific, Scotland, UK), according to the manufacturer’s instructions, and particle concentration was determined by nanoparticle tracking analysis (NTA) (ZetaView, Particle Metrix, Meerbusch, Germany). Experiments were performed in triplicate on 3 different days. From this optimisation, it was determined that fractions 5–8 will be collected as these were shown to contain the highest concentration of EV sized particles free from protein contaminants (Supplementary Figure S1).

### 2.6. Hormone Treatment of RL95-2 Cells

This experiment was designed to determine the hormonal effect on EVs produced by endometrial cells. RL95-2 cells were cultured in T75 flasks with complete DMEM culture media until ~60/70% confluent. The media was changed after being washed using DPBS without Ca^2+^ and Mg^2+^ 3 times before being incubated with phenol red free DMEM culture media with charcoal stripped FBS (10%) to adapt similar conditions and avoid possible hormone-like (estrogenic) activity of phenol red for 24 h. The appropriate hormone treatment was then added to the cells in phenol red-free DMEM culture media at either a final concentration of 10 nM of progesterone (water soluble, Sigma-Aldrich, Dorset, UK) (P4) or 10 nM of estrogen (water soluble, Sigma-Aldrich, Dorset, UK) (E2) or estrogen (10 nM) plus progesterone (100 nM) (E2P4), as described before [18,19]. A control group containing equal amounts of the solubility complexing agent, cyclodextrin, found within the hormone mixtures was also performed. Following a 24 h incubation period, conditioned media was collected, and differential centrifugation was performed, followed by concentration and size exclusion chromatography, as described above. Samples were then analysed for particle concentration via NTA and protein concentration using the BCA assay. Experiments were performed in triplicates on 3 different days.

### 2.7. Transmission Electron Microscopy (TEM)

After SEC, 100 µL of the concentrated EVs were mixed with 100 µL of 4% paraformaldehyde (Polyscience, Warrington, PA, USA) to fix the EVs. On the day of analysis, 20 µL of an EVs droplet was covered by a 200-mesh formvar and carbon coated grid (Electron Microscopy Sciences). The EVs droplet was incubated for 20 min at room temperature. After 20 min, the grid was tapped on filter paper to remove excess PBS. Later the grid was rinsed once by gently touching the surface of the grid onto 50 µL of ddH2O droplet and tapped on filter paper to remove the excess water. For negative staining, the grid was transferred onto a droplet of 4% of uranyl acetate (21447 25, Polyscience, Warrington, PA, USA) for 5 min incubation at room temperature. Samples were observed with a JEM 1400 transmission electron microscope (JEOL Ltd., Tokyo, Japan) at 80 kV, and digital images were acquired with a numeric camera (Morada TEM CCD camera, Olympus, Germany).

### 2.8. Proteomic Analysi—Label-Free Liquid Chromatography Tandem Mass Spectrometry (LC-MS/MS)

EV samples (100 µL each) were transported to the Proteomic Core Facility, University of Tartu, Estonia in dry ice for proteomic analysis. An amount of 1 µg of protein was injected onto an Easy-nLC 1000 system (Thermo Scientific, Scotland, UK). The sample was eluted at 250 nL/min from the trap to a 75 µm ID × 50 cm emitter-column (New Objective, Littleton, CO, USA) packed with C18 material (3 µm, 300 Å particles, Dr Maisch, Ammerbuch, Germany). The separating gradient was 2–35% B for 60 min and 40–100% B for 5 min (A: 0.1% formic acid (FA), B: 80% Acetonitrile (CAN) + 0.1% FA). Eluted peptides were sprayed onto a Q Exactive Plus (Thermo Fisher Scientific, Waltham, MA, USA) quadrupole-orbitrap mass spectrometer (MS) using nano-electrospray ionization at 2.4 kV (applied through liquid-junction). The MS was operated with a top-5 data-dependent acquisition strategy. Briefly, one 350–1400 *m*/*z* MS scan at a resolution setting of R = 70,000 at 200 *m*/*z* was followed by five higher-energy collisional dissociation fragmentations (normalized collision energy of 26) of the 5 most intense ions (z: +2 to +6) at R = 17,500. MS and MS/MS ion target values were 3 × 10^6^ and 5 × 10^4^, respectively, with a 50 ms injection time. Dynamic exclusion was limited to 40 s.

### 2.9. Proteomic Data Analysis

Mass spectrometric raw files were processed with the MaxQuant software package (versions 1.6.15.0 and 2.0.3.0). Methionine oxidation, asparagine and glutamine deamidation and protein N-terminal acetylation were set as variable modifications, while cysteine carbamidomethylation was defined as a fixed modification. Label-free protein quantification (LFQ) was enabled with LFQ and the protein minimum ratio count set to 1. The search was performed against *Homo sapiens and Bos taurus* reference proteomes, using the tryptic digestion rule. The peptide-spectrum match and protein false discovery rate (FDR) were kept below 1% using a target-decoy approach. All other parameters were default. Protein-coding of significant altered proteins (SAPs) in Evs were identified based on a fold change cut-off of 1 and a *p*-value cut-off of 0.05. An analysis of SAPs was performed in R studio (version 4.2.1) using the Bioconductor “DEP” package (version 3.1.5). Identified duplicates and contaminants were excluded from the list of each sample, and only proteins detected in at least two of the replicates were considered. After a literature review of these proteins enriched in the E2P4 group, 17 were selected for their functional relevance in implantation success and were divided into three groups depending on their involvement in embryo implantation, endometrial receptivity, and embryo development (Table 1). Gene Ontology (GO) and Kyoto Encyclopedia of Genes and Genomes (KEGG) pathway enrichment analyses were performed by the “clusterProfiler” R package (Version 4.4.4). Volcano plots, GO terms, and KEGG pathways were visualized by the “ggplot2” R packages (version 3.3.6). The data sets used and/or analysed during the current study are not currently publicly available, accession numbers have not yet been obtained; at the time of submission, they will be provided during review.

### 2.10. Statistical Analysis

GraphPad Prism version 9.2.0 was used for all statistical analysis. The comparison of particle concentration between the hormonal conditions and the control was analysed with repeated measure one way ANOVA. When comparing the different sizes and the different treatment groups, a two-way ANOVA with multiple comparisons was performed. All data presented as mean plus/minus standard error (mean ± SE). In all analyses, *p* < 0.05 was considered to be statistically significant.

## 3. Results

### 3.1. Characterisation of EVs Released from RL95-2 Cells

**Figure 1:**
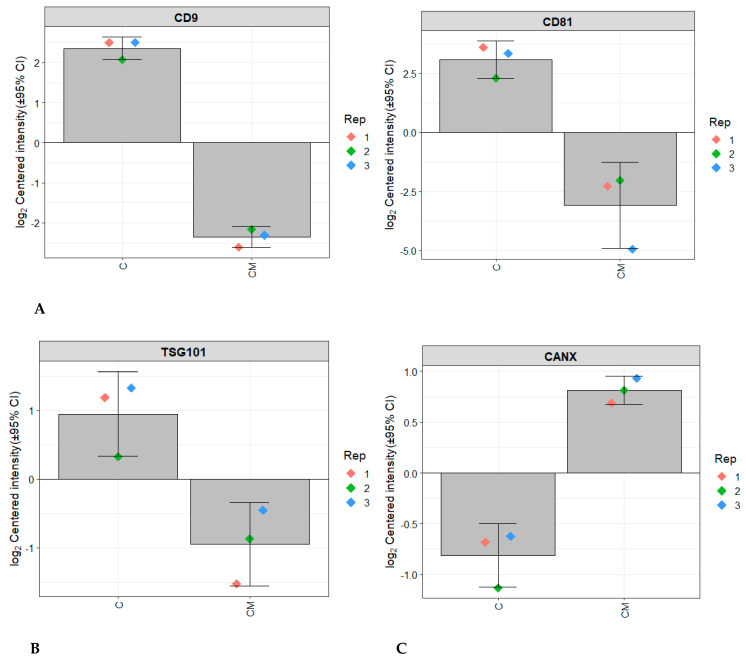
Protein content−based EV characterisation. Graphs representing the log2 centred intensity of various protein markers in the control, as well as the conditioned media control, validating the presence of EVs according to the MISEV guidelines. (**A**) represents is the intensity of the markers CD9 and CD81 to fulfil the MISEV criteria for markers of transmembrane or GPI-anchored proteins associated to plasma membrane and/or endosomes. (**B**) represents is the intensity of TSG101 to fulfil the MISEV criteria for a marker of cytosolic protein recovered in EVs. (**C**) represents is the intensity of the marker CANX to fulfil the MISEV criteria for a marker of a major components of non-EV co-isolated structures.

**Figure 2:**
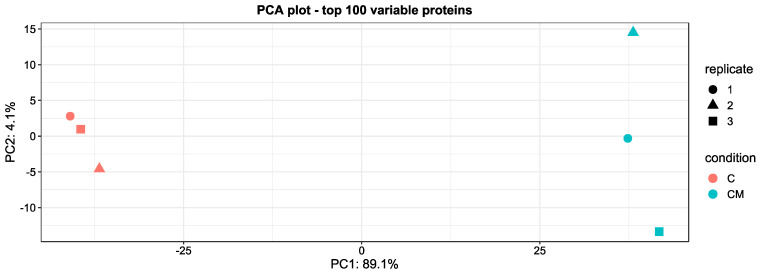
Principal component analysis (PCA) of top 100 variable proteins in the study groups. With C representing the EVs from the control group and CM representing the control conditioned media group. *n* = 3 for each experimental condition.

**Figure 3:**
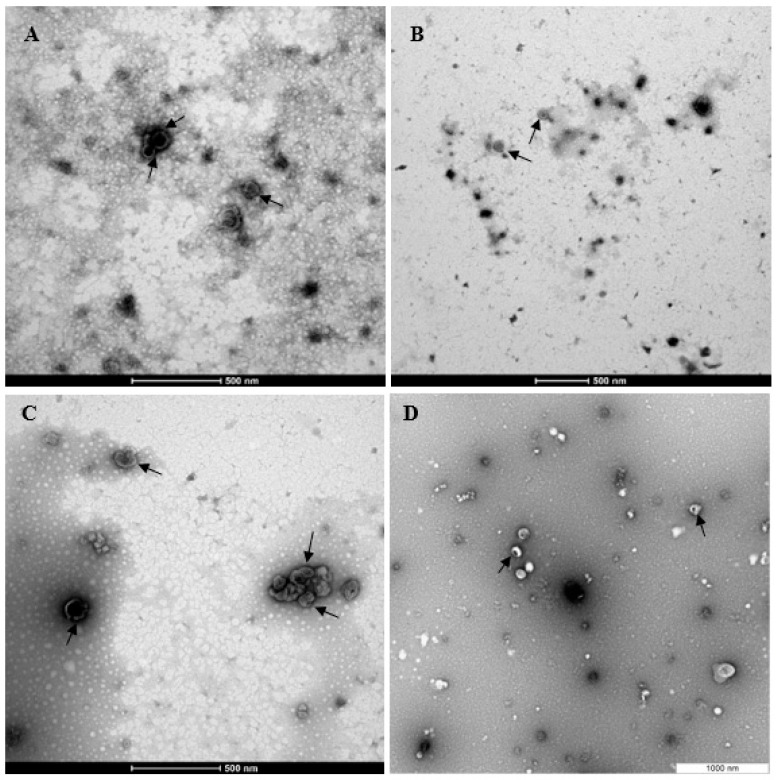
TEM Image based characterization of endometrial EVs from the different hormone treatment groups. Representative TEM images of endometrial EVs from control (**A**), P4 (**B**), E2 (**C**), and E2P4 (**D**). Scale bars: 500 nm (**A**–**C**); 1000 nm (**D**).

Purified EVs were shown to be positive for markers of transmembrane proteins associated with the EV plasma membrane CD9 and CD81 which were not seen in the conditioned media control (Figure 1A). EVs isolated from the control group conditions were also positive for TSG10, a marker of cytosolic protein recovered in EVs (Figure 1B) and negative for the negative marker Calnexin (CANX), a major component of non-EV co-isolated structures (Figure 1C). The PCA analysis also showed clear clustering between the control EV group and the control CM group (Figure 2). Transmission electron microscopy confirmed that EVs were intact, spherical in morphology, and with the expected EV size range within all four experimental conditions (Figure 3). Combined, these results fulfil the minimal information for studies of extracellular vesicles (MISEV) criteria and demonstrate that EVs have been successfully isolated from the endometrial conditioned media.

### 3.2. Concentration and Size Distribution of EVs Released from Hormone-Treated RL95-2 Cells

**Figure 4:**
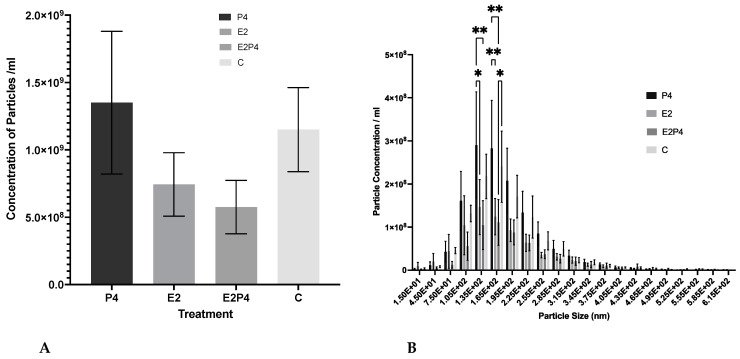
Concentration and size distribution of EVs released from RL95-2 hormone-treated cells. (**A**) The bars represent the mean concentration (particles/mL) ± SEM of EV sized particles from the SEC fractions 5–8. The treatment groups include estrogen (E2), progesterone (P4), estrogen plus progesterone (E2P4), and the control. Data were from 3 biological replicates and 3 technical replicates from each group. (**B**) The bars represent the mean concentration of the indicated particle size ± SEM from the SEC fractions 5–8. The significant difference is marked with 1 asterisk symbol (*) to denote *p* ≤ 0.05 and 2 asterisk symbols (**) to denote *p* ≤ 0.01. Data are from 3 biological replicates and 3 technical replicates.

To assess the effect of the different menstrual cycle hormones on EV production, a comparison of the concentration of EVs produced from estrogen (E2), progesterone (P4), or estrogen plus progesterone (E2P4)-treated cells were compared to a control group. The addition of the hormones in the three different treatment groups showed no difference between the amount of EV sized particles produced (Figure 4A). A size comparison analysis of the concentration of particles in the different size classes of the particles was also produced and the different treatment groups were compared (Figure 4B). A significant difference can be seen between the 135 nm and 165 nm size groups, with more EV sized particles being released by the P4-treated group compared to the E2 group or the E2P4 group.

### 3.3. Comparative Proteomic Analysis of Endometrial EVs under Hormonal Treatment

**Figure 5:**
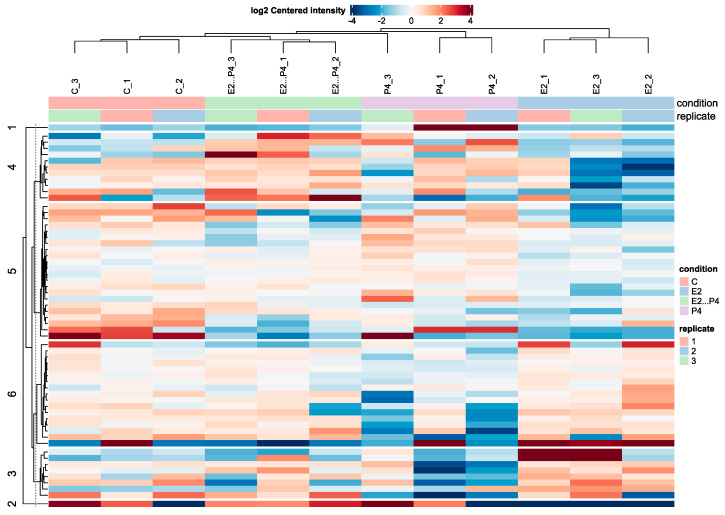
Heatmap shows the log2 centred intensity of all significant proteins with the data centred per protein. The rows represent all significant proteins and are clustered in 6 groups by k-means clustering (numbers on the left). C (control group), E2 (estrogen group), E2…P4 (estrogen and progesterone group), and P4 (progesterone group). Colours represent the abundance of significantly altered proteins (red: higher; blue: lower).

**Figure 6:**
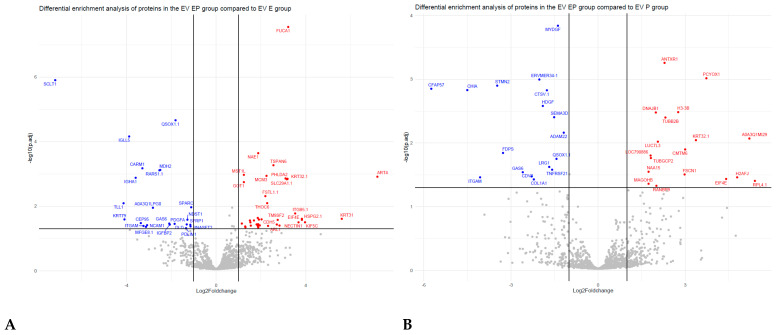

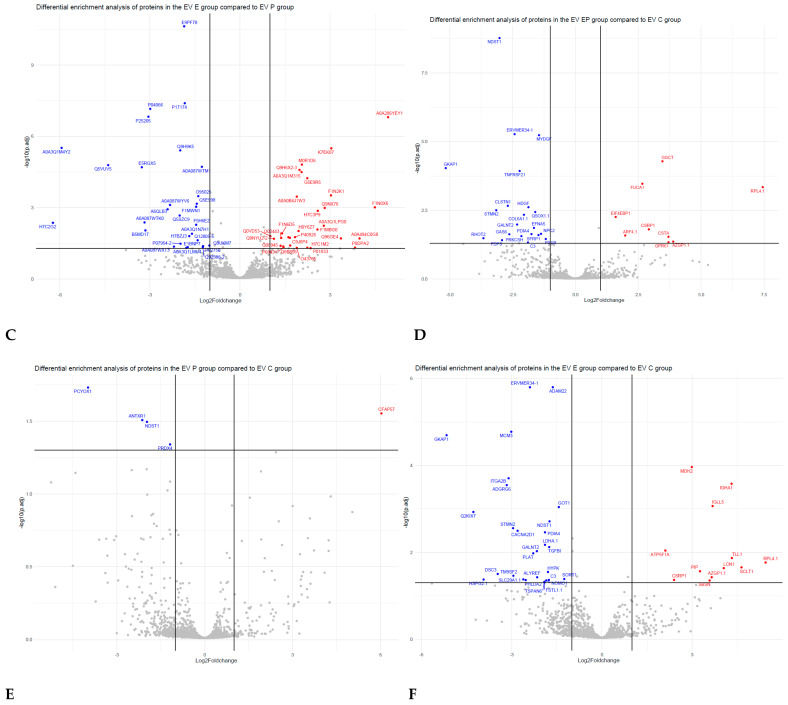
Differential enrichment of EV proteins in different comparison groups. (**A**) Volcano plot of the differentially expressed proteins in the estrogen plus progesterone supplemented groups (E2P4) compared to the estrogen group (E2); (**B**) estrogen plus progesterone supplemented groups (E2P4) compared to the progesterone group (P4); (**C**) estrogen supplemented groups (E2) compared to the progesterone group (P4); (**D**) estrogen plus progesterone group (E2P4) compared to the control (C); (**E**) progesterone (P4) compared to the control (C); and (**F**) estrogen (E2) compared to control. Adjusted *p* values (−log10) are plotted against the fold changes (log2). Red dots represent enriched proteins whereas blue dots represent depleted proteins in the respective comparisons.

A heatmap of the log2 centred intensity of all significant proteins with the data centred per protein from the four experimental conditions showed a clear grouping of the four experimental conditions with six cluster groups, showing clear varied intensities between the EV isolated groups (Figure 5). There were 60 significant altered proteins (SAP) identified from the differential enrichment of EV proteins from the E2P4 group compared to E2 (Supplementary Table S1), 35 SAP identified from differential enrichment of EV proteins from the E2P4 group compared to the P4 group (Supplementary Table S2), 50 SAP identified from differential enrichment of EV proteins from E2vP4 (Supplementary Table S3), 31 SAP identified from the differential enrichment of EV protein from E2P4vC (Supplementary Table S4), 41 SAP identified from the differential enrichment of EV protein from E2vC (Supplementary Table S5), and five SAP identified from the differential enrichment of EV protein from P4vC (Supplementary Table S6). In the E2P4 group compared to the E2 group, 37 SAP were upregulated and 23 were downregulated. In the E2P4 group compared to the P4 group, 18 SAP were upregulated and 17 were down regulated. Volcano plots of these result show a direct indication of SAP (Figure 6). After a literature review of these proteins enriched in the E2P4 group, 17 were selected for their functional relevance in implantation success and were divided into three groups depending on their involvement in embryo implantation, endometrial receptivity, and embryo development (Table 1).

**Table 1:** Proteins differentially enriched in the E2P4 receptive group EVs related to implantation success.

UniProt Accession	Gene Name	Protein Description	Evidence	Reference
	**Embryo implantation**			
Q6URK6	CDH5	Cadherin-5	CDH5 has been shown to be expressed in the late proliferative phase in the endometrium. A mouse in vitro study has demonstrated that the expression of CDH5 in trophectoderm helps to facilitate embryo implantation.	[20,21]
F1MER7	HSPG2	Basement membrane-specific heparan sulfate proteoglycan core protein	Shown to be an abundant cargo of the luteal phase EVs and have a specific role in embryo implantation. HSPG2 within the uterine epithelium has been shown to facilitate trophoblast attachment and adhesion.	[19,22]
F6RAG5	KIF5C	Kinesin heavy chain isoform 5C	Gene expression increased in embryo-endometrium contact co-cultures, as well as being shown to play a role during different morphogenetic processes in early embryo development.	[23,24]
A0A452DJ21	EIF4E	Eukaryotic translation initiation factor 4E	The expression if EIF4E has been examined in porcine endometrium during implantation, with truncated EIF4E shown to particularly regulate protein synthesis during conceptus attachment at the time of implantation.	[25]
Q58D84	FSTL1	Follistatin-related protein 1	Identified in menstrual fluid samples and is a common inflammatory and repair protein.	[26]
A6QLB3	ITGA2B	Integrin subunit alpha 2b	ITGA2B has been identified during the stages of bovine trophectoderm cell migration and fusion with the uterine epithelial cells.	[27]
Q9NRN7	AASDHPPT	l-aminoadipate-semialdehyde dehydrogenase-phosphopantetheinyl transferase	Found to be upregulated in the endometrium during pregnancy compared to genes during the estrous cycle.	[28]
	**Endometrial receptivity**			
A0A452DJE0	ACE2	Angiotensin-converting enzyme 2	ACE2 within the female reproductive system is abundantly expressed and is responsible for generating angiotensin, which has been shown to stimulate ovarian follicle growth, ovulation, and oocyte maturation, and is within the human endometrium.	[29]
H7BZJ3	PDIA3	Protein disulfide-isomerase A3	PDIA3 upregulated in fertile endometriums during secretory phase as compared to proliferative phase.	[30]
P00750	PLAT	Tissue-type plasminogen activator	In the reproductive system, PLAT mediates tissue remodelling required for ovulation and endometrial receptivity.	[31]
C9JPV1	SLC6A6	Sodium- and chloride-dependent taurine transporter	SLC6A6 gene expression has been shown to be greater in cow endometria in the large preovulatory follicle compared to the small preovulatory follicles. This gene is thought to have a role in amino acid metabolism which is linked to the receptive state of the endometrial tissue.	[32]
A0A087WYV6	TSPAN6	Tetraspanin-6	Found in endometrial exosomes and thought to have functional and regulatory roles.	[33]
M0R1D6	DNAJB1	DnaJ homolog subfamily B member 1	One of many novel genes identified and associated with the biological process of menstrual cycle.	[34]
D6RDI2	LUC7L3	Luc7-like protein 3	Upregulated in human endometrial sample in women who exhibited implantation failure.	[35]
A0A3Q1LMU4	INHBB	Inhibin beta B chain	Known marker gene of decidualization.	[36]
	**Embryo development**			
P04066	FUCA1	Tissue alpha-l-fucosidase	FUCA1 was seen to be in higher abundance at elongation, as well as upregulated during day 15 of the cycle compared to day 7.	[37,38]
P00338	LDHA	l-lactate dehydrogenase A chain	When LDHA is inhibited, histone lactylation level is reduced and an impaired rate of development of pre-implantation mice embryos is seen.	[39]

**Figure 7:**
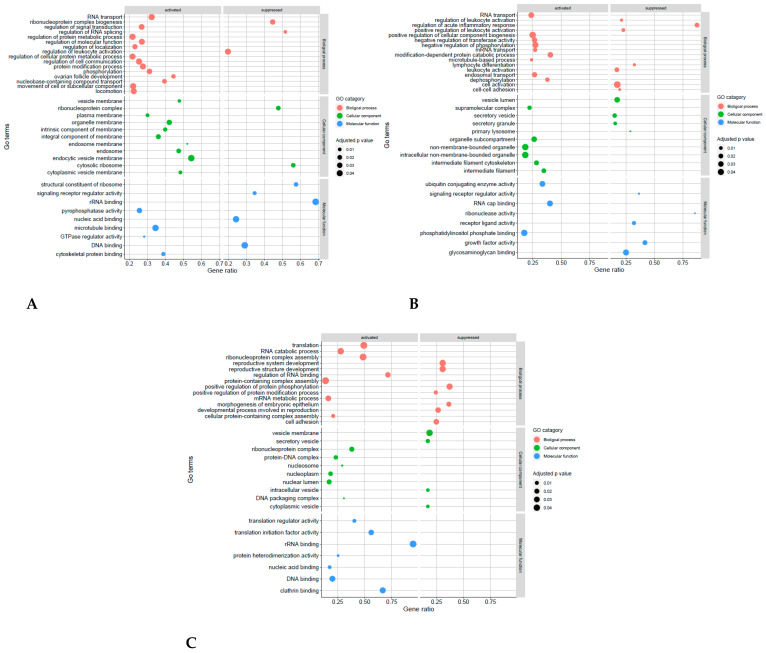
GO enrichment of SAP in the different comparison groups (**A**) E2P4 v E2, (**B**) E2P4 v P4, (**C**) E2vP4. The size and colour of points represents the GO category and the q-values respectively.

**Figure 8:**
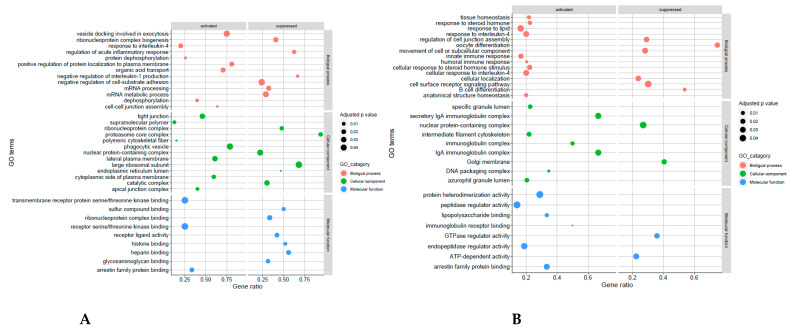

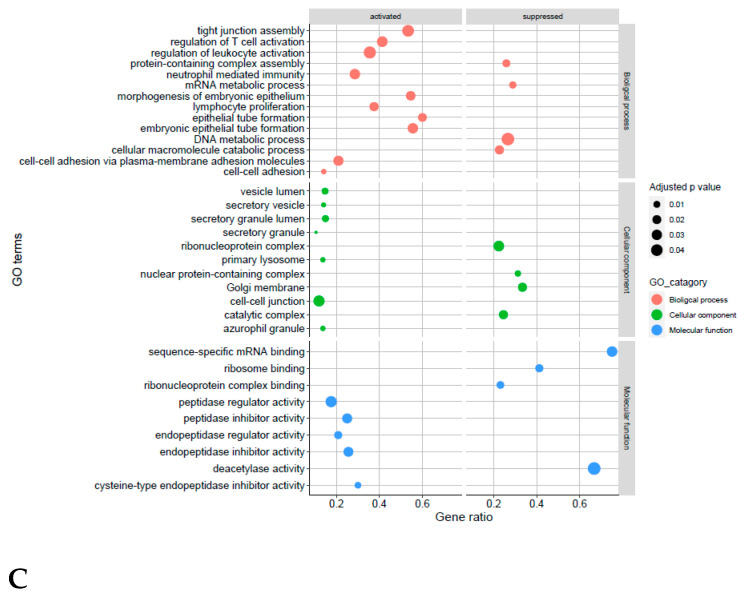
GO enrichment of SAP in the different comparison groups (**A**) E2P4 v C, (**B**) E2 v C, (**C**) P4 v C. The size and colour of points represents the GO category and the q-values respectively.

**Figure 9:**
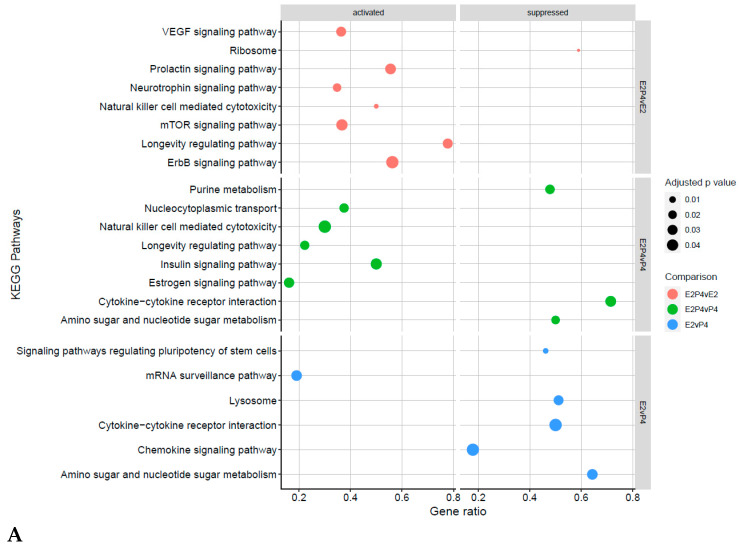

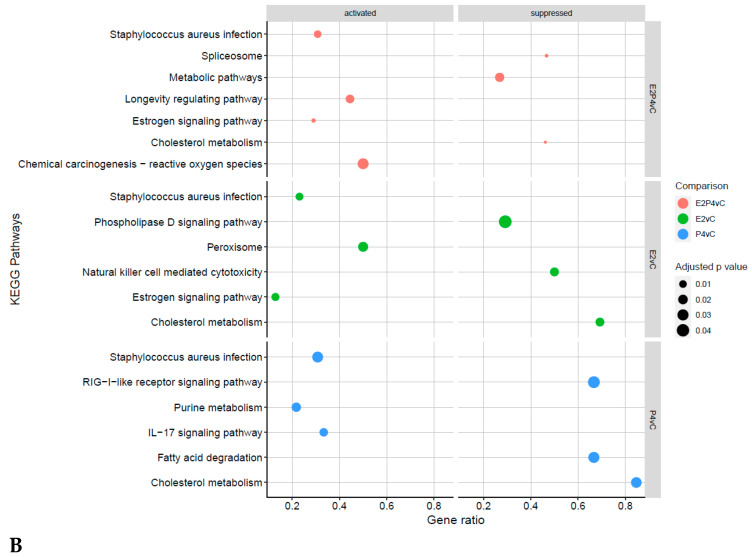
KEGG Pathway analysis of SAP in the comparison groups and control. (**A**) Pathway analysis of SAP in the comparison groups, the size and colour of points represents the *p*-values and the comparison groups respectively. (**B**) KEGG Pathway analysis of SAP in the treatment groups versus the control, the size and colour of points represents the *p*-values and the comparison groups respectively.

To further analyse the biological function of DEGs, GO terms and KEGG pathway enrichment analyses were performed on the three comparison groups (E2P4vE2, E2P4vP4, and E2vP4) and the controls (E2P4vC, E2vC, and P4vC) (Figure 7, Figure 8 and Figure 9). GO function analysis was conducted first. Regarding the biological process (BP), in E2P4vE2, the enriched SAP were involved in the regulation of cell communication, RNA transport, the movement of the cell or subcellular component, and protein modification (Figure 7A). In E2P4vP4, the SAP were enriched in endosomal transport, dephosphorylation, RNA transport, and mRNA transport (Figure 7B). In E2vP4, the SAP were enriched in protein-containing complex assembly, RNA catabolic process, translation, and mRNA metabolic processes (Figure 7C). In terms of molecular function (MF), in the E2P4vE2 group, enrichment was seen in cytoskeletal protein binding, microtubule binding, and GTPase regulator activity (Figure 7A). In E2P4vP4 the SAP were enriched in phosphatidylinositol phosphate binding, RNA cap binding, and ubiquitin conjugating enzyme activity (Figure 7B). In E2vP4, enrichment was seen in rRNA binding, DNA binding, and translation regulator activity (Figure 7C).

KEGG pathway analysis showed various pathways enriched in the comparison groups and control (Figure 9A,B). In E2P4vE2, enriched pathways included the mTOR signalling pathway, prolactin signalling pathway, and ErbB signalling pathway. In E2P4vP4, pathways that were intensified included the insulin signalling pathway, natural killer cell mediated cytotoxicity, and estrogen signalling pathway. In E2vP4, supressed pathways included cytokine-cytokine receptor interaction, lysosomes, and chemokine signalling pathways.

## 4. Discussion

A successful pregnancy requires a receptive endometrium and a good quality embryo and optimum communication between these two entities. Locally, this complex process involves mutual communication between the incoming embryo and the endometrium in the uterine milieu [40,41,42,43]. Recently, it has been suggested that EVs regulate early embryo development as it reaches the uterus, in addition to priming the endometrium for embryo implantation [5,44,45]. The Evs lipid-bilayer protects the cargo, including proteins, from degradation, making them highly stable. However, a complete understanding of EV and their cargo’s role in mediating implantation has not been demonstrated. This study comprehensively evaluates the proteome of EVs secreted by RL95-2 cells, an analogue of receptive endometrial epithelial cells, under a hormone stimulation mimicking the menstrual cycle to help begin to define novel biomarkers of endometrial receptivity and implantation success.

RL95 has been used in a number of papers and is a well-established model of a receptive endometrium [46,47,48]; additionally this cell line has been used in many implantation and adhesion related assays [49,50,51]. Thus, by using this cell line, we can provide novel insights into the complex process of human embryo implantation regulation. Via this in vitro modulation, discrepancies and variations between endocrinologic milieu Via among the individuals in a population can be avoided. An in vitro endometrial cell culture-based model, despite its simplicity, can offer a simple and a general characterisation and understanding of the EV proteome. Interestingly, 112 proteins were seen to be upregulated in the control EV samples compared to the conditioned medium, and of these proteins only 12 have been previously identified as exosome markers in Vesiclepedia, (ANXA6, ANXA7, ATP1A1, BSG, CD9, FLOT1, GNAS, GNB2, ITGB1, RAB7A, SDCBP, TFRC) (Supplementary Figure S2). The presence of these markers further supports their use as indicators of the enrichment of EVs. The additional 100 proteins enriched in EVs could be additional potential new markers for EVs or endometrial EVs, such as integrin alpha-3, a membrane protein that regulates multiple biological processes, including cell adhesion, cell differentiation, and cell migration.

Moreover, the results indicate that the effects of all the hormone experimental conditions when compared to the control had no statistically significant differences in terms of EV concentration in vitro. A similar in vitro study showed results comparable to ours in that EVs were secreted in numbers independent of hormonal regulation, with similar concentrations seen between the estrogen and progesterone-treated groups [19]. However, these results and ours contradict those of an in vivo study which demonstrated that P4 increased the concentration of EVs in the uterine lumen [52]. However, it is important to note the different cell lines used when comparing studies, as well as the complex microenvironmental influences from different in vivo models.

In terms of EV size distribution, it was observed that the EV fractions from all four treatment conditions were within the expected EV size range but also, interestingly, there was a significant difference seen between the 135 nm and 165 nm size groups with more EV being released by the P4-treated group compared to the E2 group or the E2P4 group. This could suggest that higher amounts of EVs within this size range in the progesterone group, the hormone most prominent during the secretory phase when the endometrium is fertile, is due to an increase in communication signalling occurring, either sending a signal to the incoming embryo or in an autocrine way for the endometrium to prime itself for the incoming embryo. Alternatively, the larger size could be the presence of more cargo due to induction of secretory activity of the epithelial cells by the progesterone, which indirectly improves communications. However, when interpreting these results, it is Important to remember the limitations of NTA, the lack of specificity means differentiation between non-EV-structures, such as lipoproteins or residual reagent residues, could influence these results. Thus, only limited conclusions can be drawn from this method alone.

### 4.1. Receptive Phase Endometrial Cell EVs Were Enriched with Proteins Involved in Embryo Implantation

Proteomic assessment by the mass spectrometry of EVs allows a broader analysis to be performed. It has previously been shown that EVs derived from the endometrium can signal to the embryo to promote implantation. However, their composition, function, and influence on the uterine microenvironment and embryo remain unknown. Through proteomic analysis, we identified some key proteins that were significantly increased in E2P4-treated endometrial cell EVs compared to both E2 or P4 endometrial cell derived EVs, and these proteins may have a specific role in embryo implantation. Several of these proteins (including Cadherin-5 “CDH5”, heparan sulfate proteoglycan core protein “HSPG2”, Kinesin heavy chain isoform 5C “KIF5C” and Eukaryotic translation initiation factor 4E “EIF4E”) have previously been identified in various embryo implantation models. Cadherin are cell adhesion molecules of which there are more than eighty members and are key regulators of cell adhesion, sorting and invasion [20]. In the current study, CDH5, was shown to be upregulated in EVs from the E2P4-treated cells, and has similarly been shown to be expressed in the late proliferative phase in endometrial explants [53] as well as endometrial mesenchymal stromal cells [54]. In addition an in vitro mouse study demonstrated that expression of CDH5 in the trophectoderm facilitates embryo implantation [21]. Such studies suggest that the upregulation of CDH5 in EVs specifically in the stimulated receptive period could be contributing to embryo implantation. Of further interest to the developmental progress of pregnancy is our identification of Perlecan (HSPG2) as intrinsic to the EV proteome. This molecule has been shown to be highly regulated at the time of blastocyst apposition and attachment, with previous studies demonstrating this role and its presence in the uterine epithelium [55,56]. All this data point towards the role of the receptive endometrium in contributing to the implantation interface through the release of EVs, potentially by signalling to, and preparing the endometrium to support implantation during this specific period dictated by hormones.

### 4.2. Receptive Phase Endometrial EVs Were Enriched in Proteins Involved in Embryo Development

The analysis of our dataset from the E2P4 EVs showed further proteins to be increased that are important in embryo development. These include proteins (including, “FUCA1” tissue alpha-L-fucosidase, and “LDHA1” l-lactate dehydrogenase A chain) that have previously been identified in various models assessing embryonic development. For example, the protein FUCA1 was seen in higher amounts in the EV isolated from the receptive phase induced endometrium condition in our study. Interestingly this protein was one of many that have also been seen at a higher level in the uterus of deer while embryos were at elongation rather than during diapause [37]. This suggests that changes occur upon embryo elongation in the uterine environment to support early development and survival, and perhaps our study suggests that endometrial EVs are contributing to transporting these proteins. Additionally, LDHA was seen to be upregulated in the E2P4 group and has also been shown to have a role in embryo development. Within pre-implantation, embryo glycolysis is regulated by LDHA, with LDHA catalysing the conversion of pyruvate to lactate. It has been shown that when LDHA is inhibited, the histone acetylation level is reduced and an impaired rate of development of pre-implantation mice embryos is seen [39]. Taken together this data suggest that endometrial EVs during the receptivity period could contain proteins associated with embryo development and that these are mutually communicated between the embryo and mother.

### 4.3. Receptive Phase Endometrial EVs Were Enriched in Proteins Involved in Endometrial Receptivity

On further analysis of our dataset, we observed an increase in proteins within E2P4 EVs compared to both E2 or P4 that are important in endometrial receptivity. These include proteins (including and “PLAT” tissue-type plasminogen activator, angiotensin-converting enzyme “ACE2” and inhibin beta B chain “INHBB”), that have previously been identified in various endometrial receptivity models. ACE2 has been found previously to be highly expressed within the reproductive system and is responsible for generating angiotensin using precursor peptides produced locally or taken from the circulation. Angiotensin within various mammalian species is seen to stimulate ovarian follicle growth, ovulation and oocyte maturation, and, within the human endometrium, it is seen at its highest levels during the window of implantation [57]. In addition, it has previously been discovered in airway epithelial cells that estrogen stimulated ACE2 expression, suggesting that the expression seen here also could also be mediated by steroid hormones [58]. However, the precise role of ACEs in the human endometrium is yet to be revealed and more evidence is required. In addition to ACE2, the protein PLAT was also seen to be upregulated in the stimulated receptive phase EVs. Again this protein has also been suggested to have a role in endometrial receptivity with PLAT seen to be involved in the extracellular matrix degradation essential for embryo adhesion and placental angiogenesis [31]. Finally, an increase in INHBB is seen in the receptive phase stimulated EVs. It has been previously reported that INHBB gene expression within the human uterus is related to the onset of decidualization [59], with its expression increasing in human endometrial stromal cells during decidualisation in vitro [60]. In addition to this, a reduced expression and reduced decidualization has been shown in ectopic pregnancies, implying that INHBB expression within human pregnancy is dependent on decidualisation [60]. These studies support the theory that the proteins found within the endometrial EV within the receptive phase have a role in endometrial receptivity too.

### 4.4. Receptive Phase Endometrial EVs GO Terms and KEGG Pathways Are Associated with Receptivity and Implantation Success

GO and KEGG analyses reinforced the above findings and identified key pathways associated with receptivity and implantation success in the receptive group. In the E2P4 group, in both the E2P4vE2 and E2P4vP4 group, key pathways in immune modulation, specifically natural killer cells, were activated. It has been previously reported that the levels of NK cells change during the menstrual cycle and pregnancy [61], with increases seen during the secretory phase followed by a peak during the early stages of pregnancy [62]. There are various publications that support the theory that NK cells help in the regulation of pregnancy establishment and maintenance through many mechanisms, such as cytokine production that directly influence trophoblast growth [63], in addition to the vascularization and implantation of the decidua [64], immune modulating protein production that has a role in regulation of the immune response at the maternal-foetal interface [65], as well as the regulation of trophoblast invasion by cell-mediated cytotoxicity [63]. Despite this activation, cytokine-cytokine receptor interaction was shown to be supressed in the E2P4 group. This contradiction has been suggested to derive from the concept that endometrial NK cells are special immature cells that await pregnancy, with the NK cells lacking NK-specific functional phenotype and activity, such as cytokine secretion and cytotoxicity [66]. Hence the activation and suppression of these pathways reflects the phases stimulated in this model, with no influence of pregnancy or embryo stimulus.

In addition to the immune modulation, the KEGG pathway analysis also revealed many pathways known to support implantation in the E2P4 group, including the mTOR signalling pathway and VEGF signalling pathway. The mTOR pathway is known to play a role in cellular functions, such as cell proliferation, differentiation, apoptosis, autophagy, and decidualization [67], all of which are requirements for modulating a receptive endometrium. Similarly to what is seen in our data, it has previously been shown that mTOR increases during the implantation window in mice, with its role in receptivity further highlighted in a study in which an intrauterine injection of an inhibitor of mTOR, rapamycin, compromised the endometrial receptivity in mice [68]. Like mTOR, VEGF is also well reported to have a role in improving endometrial receptivity and facilitating the interactions between the embryo and the endometrium. VEGF, is an endothelial-specific mitogen in vitro, and is a main physiological and pathological angiogenesis regulator in the endometrium [69]. Within rodents, it has been shown that VEGF regulates endometrial vascular permeability and endothelial cell proliferation at the implantation site, indicating a key role of the VEGF pathway in embryonic-endometrial interaction [70,71]. It is well established that expression of VEGF dynamically changes within the oestrus cycle period and has been shown to be highly expressed in the endometrium in the middle stage of secretion, which mimics results seen in this study with the pathways activated in the E2P4 EV group. None of these pathways were seen to be activated in the E2- or P4-only-treated endometrial cell EVs, suggesting that the EV proteome is changing during the menstrual phases but a mechanism of how/if these proteins physiologically affect the endometrium/embryo requires further investigation.

A limitation and implication of interpreting this proteomic data, however, lies in the use of the proteomic method of shotgun label free proteomics. One issue that this approach highlights is that only the most abundant proteins are identified due to limitations in detection methods; hence, further proteins could be identified as being altered in the various groups, had an alternative method been used. This might have identified other pathways or roles that endometrial EVs contribute to during the menstrual cycle. Additionally, the results relate to the sample’s total proteome, and it cannot be determined if they are located on the EVs cell surface or exist as cargo. Instead, the results are of a complex total EV proteome and further investigation via other means, such as antibody microarray, would be needed to understand their precise origin (i.e., cargo, surface, or both). Thus, whether these proteins are surface bound or cargos of EVs, and hence how, and if, they are of physiological significance in embryo-maternal signalling, remains an open question.

## 5. Conclusions

In summary, findings of the present study provide a new insight into the characteristics of EV from endometrial cells under hormonal control. Results from this study have provided new information on the effect menstrual cycles have on EV production, demonstrating a contribution of endometrial-derived EVs to endometrial-embryo cross talk, showing an effect even without the known contributions of other players, such as spermatozoa, oocyte, and the developing embryo in an environment that is constantly changing and sending and receiving signals. Here the total proteome of endometrial EVs has been shown to be regulated by estrogen and progesterone, as physiologically relevant to the phases of the menstrual cycle. Significantly, our study shows that endometrial EVs within the receptive phase of the menstrual cycle consists of proteins associated with embryo implantation, endometrial receptivity, and embryo development, and key pathways identified in immune modulation and implantation processes. The application of this knowledge may allow EVs to be exploited as an endometrial tissue biopsy alternative and have a range of applications, such as monitoring the window of implantation, identifying the underlying aetiology of recurrent implantation failure, better prediction and increased implantation success in IVF, and enabling the design of non-hormonal-based contraceptives.

## Data Availability

The data that support the findings of this study is available from the corresponding author upon reasonable request. The mass spectrometry proteomics data have been deposited to the ProteomeXchange Consortium via the PRIDE [73] partner repository with the dataset identifier PXD039764.

## Supplementary Materials

The following supporting information can be downloaded at: https://www.mdpi.com/article/10.3390/biom13020279/s1, Figure S1: Isolation of extracellular vesicles sized particles secreted by the endometrial cell line RL95-2 using differential centrifugation and size exclusion chromatography; Figure S2: Ven diagram of proteins enriched in the Control EV group versus the conditioned media control compared to the top 100 Vesiclepedia EV markers; Table S1: Differential enrichment of EV proteins of E2P4 Group compared to E2; Table S2: Differential enrichment of EV proteins of E2P4 Group compared to P4; Table S3: Differential enrichment of EV proteins of E2 Group compared to P4; Table S4: Differential enrichment of EV proteins of E2P4 Group compared to C.; Table S5: Differential enrichment of EV proteins of E2 Group compared to C; Table S6: Differential enrichment of EV proteins of P4 Group compared to C. Reference [72] is cited in the supplementary materials.
