# Supplementary material for: The Extracellular Vesicles Proteome of Endometrial Cells Simulating the Receptive Menstrual Phase Differs from That of Endometrial Cells Simulating the Non-Receptive Menstrual Phase
Source: Biomolecules. 2023 Feb 2;13(2):279. doi: 10.3390/biom13020279 (PMC9953153; doi:10.3390/biom13020279)
Supplement: Supplementary file 1 [file biomolecules-13-00279-s001.zip › biomolecules-2181284-supplementary.pdf]

biomolecules

MDPI

Supplementary Materials

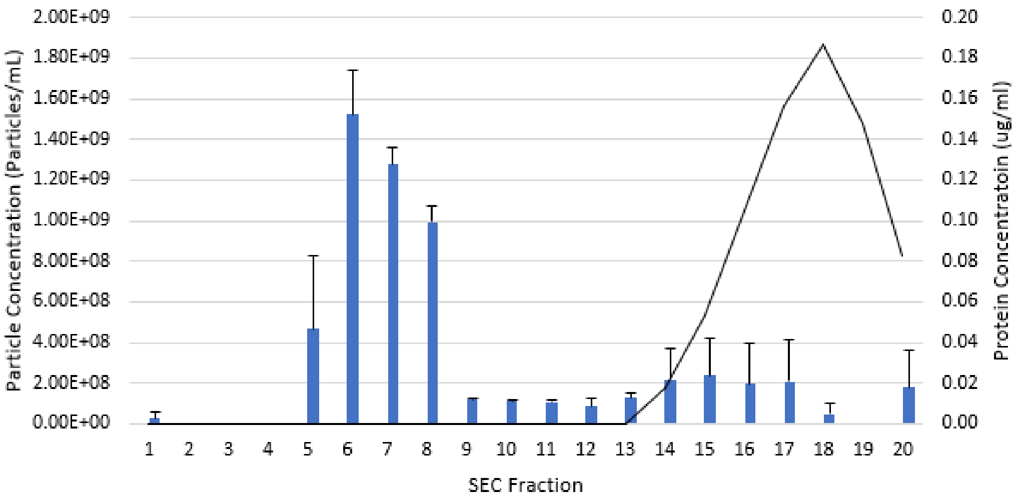

Figure S1. Isolation of extracellular vesicles sized particles secreted by the endometrial cell line RL95-2 using differential centrifugation and size exclusion chromatography. The height of the bars represents the mean concentration (particles/mL) of EVs from three biological replicates. The majority of EVs sized particles were eluted in fractions 5–8. The line graph represents the mean concentration of protein (mg/mL) in each SEC fraction from three biological replicates. Error bars represent the standard error from the mean.

Table S1. Differential enrichment of EV proteins of E2P4 Group compared to E2. Protein identified in EVs released from estrogen- plus progesterone-treated RL95-2 cells to estrogen-treated RL95-2 cells.

Uniport Accession	Gene Name	Adjusted *p* Value	Fold Change
A0A494C0G8	SCLT1	1.25×10^{-6}	−7.18
F1N0X6	TLL1	0.00812	−4.13
Q148H7	KRT79	0.0257	−4.09
A0A0B4J231	IGLL5	6.93×10^{-5}	−3.88
A0A286YEY1	IGHA1	0.00132	−3.59
Q96GE4	CEP95	0.0334	−3.36
F1MBG0	CARM1	0.00067	−3.29
P11215	ITGAM	0.0409	−3.24
Q95114	MFGE8.1	0.0437	−3.13
A0A3Q1N1J8	NCAM1	0.0388	−3.08
A0A3Q1LPG0	A0A3Q1LPG0	0.0112	−2.82
A7YW98	RARS1.1	0.00077	−2.53
P40926	MDH2	0.00076	−2.49
Q14393-5	GAS6	0.0345	−2.09
P18065	IGFBP2	0.0372	−2.07
H7BYW6	PDGFA	0.0352	−1.85
F1MM32	QSOX1.1	2.18×10^{-5}	−1.81
O00151	PDLIM1	0.048	−1.34
E9PEX6	DLD	0.0367	−1.31
A0A3Q1M688	NDST1	0.0262	−1.28
Q8N474	SFRP1	0.0377	−1.15
D6RHI9	RNASET2	0.0423	−1.14
F5GY03	SPARC	0.0108	−1.11
Q99523	SORT1	0.0345	1.15

P17174	GOT1	0.0018	1.24
Q2TV78-2	MST1L	0.00107	1.24
A0A3Q1MNL9	TGFBI	0.0424	1.3
Q17QQ1	LAMTOR3	0.045	1.31
P19858	LDHA.1	0.0277	1.51
A0A3Q1N7H1	CYRIB	0.0317	1.53
Q3SZF2	ARF4.1	0.04	1.55
H7BZJ3	PDIA3.1	0.0277	1.69
E9PAM4	PI4K2A	0.0367	1.81
Q9NRN7	AASDHPPT	0.0231	1.86
A6QLB3	ITGA2B	0.0406	1.86
O75223	GGCT	0.0362	1.88
A0A3Q1LMU4	INHBB	0.0451	1.88
Q13564-3	NAE1	0.00023	1.88
P00750	PLAT	0.026	1.91
A0A452DJE0	ACE2	0.0394	1.95
C9JPV1	SLC6A6	0.0256	2.03
Q58D84	FSTL1.1	0.00488	2.2
P25205	MCM3	0.00115	2.25
Q86W42	THOC6	0.00806	2.28
A0A087WXT3	ZNF33B	0.0408	2.32
A0A087WYV6	TSPAN6	0.00054	2.56
Q6URK6	CDH5	0.0369	2.71
F1N672	TM9SF2	0.0266	2.75
B4DZG7	ARL1	0.0398	2.83
Q53GA4	PHLDA2	0.00138	3.1
Q3ZC83	SLC29A1.1	0.00147	3.16
A6QP90	KRT32.1	0.00142	3.19
P04066	FUCA1	$2.81x10^{-8}$	3.22
A0A3Q1M5K6	ITGB5.1	0.0168	3.53
Q15223	NECTIN1	0.0316	3.68
A0A452DJ21	EIF4E	0.0265	3.83
F1MER7	HSPG2.1	0.0246	3.83
F6RAG5	KIF5C	0.0315	3.97
Q148I8	KRT31	0.0246	5.61
H7C2G2	ART4	0.00122	7.2

Table S2. Differential enrichment of EV proteins of E2P4 Group compared to P4. Protein identified in EVs released from estrogen plus progesterone-treated RL95-2 compared to progesterone-treated RL95-2 cells.

Uniport Accession	Gene Name	Adjusted *p* Value	Fold Change
A0A3Q1M4Y2	CFAP57	0.00141	−5.74
Q5VUV5	CHIA	0.00148	−4.5
P11215	ITGAM	0.0345	−4.06
E5RGX5	STMN2	0.00126	−3.47
P14324-2	FDPS	0.0143	−3.27
Q14393-5	GAS6	0.0287	−2.59
H0Y8K3	CDV3	0.0334	−2.29
P02453	COL1A1	0.0372	−2.21
Q9H9K5	ERVMER34-1	0.00101	−2.02
P51858	HDGF	0.00262	−1.9

Q5E998	CTSV.1	0.00149	−1.76
P02750	LRG1	0.0237	−1.68
O75509	TNFRSF21	0.0262	−1.58
O95025	SEMA3D	0.00394	−1.51
F1MM32	QSOX1.1	0.0177	−1.43
Q969H8	MYDGF	0.000145	−1.38
E9PF78	ADAM22	0.00688	−1.18
F5H6P7	MAGOHB	0.0435	1.74
A0A0B4J1W3	NAA15	0.0282	1.74
A0A3Q1M315	LOC790886	0.0157	1.81
Q9BSJ2-3	TUBGCP2	0.0172	1.82
M0R1D6	DNAJB1	0.00332	1.99
Q96S59	RANBP9	0.0469	2.01
D6RDI2	LUC7L3	0.00952	2.06
Q9H6X2-3	ANTXR1	0.000554	2.29
Q6B856	TUBB2B	0.00398	2.32
K7EK07	H3-3B	0.00328	2.76
C9JPH9	FSCN1	0.0311	2.98
Q9NX76	CMTM6	0.0126	3
A6QP90	KRT32.1	0.00901	3.37
F1N2K1	PCYOX1	0.000965	3.73
A0A452DJ21	EIF4E	0.0366	4.41
Q3ZBX9	H2AFJ	0.0345	4.79
A0A3Q1MI29	A0A3Q1MI29	0.00849	5.21
A0A3Q1MM65	RPL4.1	0.0392	5.4

Table S3. Differential enrichment of EV proteins of E2 Group compared to P4. Protein identified in EVs released from estrogen plus progesterone-treated RL95-2 compared to control treated RL95-2 cells.

Uniport Accession	Gene Name	Adjusted *p* Value	Fold Change
H7C2G2	ART4	0.00429	−6.23
A0A3Q1M4Y2	CFAP57	0.00000306	−5.94
Q5VUV5	CHIA	0.0000162	−4.39
E5RGX5	STMN2	0.0000199	−3.27
A0A087WTK0	PTPRJ	0.00414	−3.18
B5MD17	CBX1	0.00897	−3.15
P25205	MCM3	0.000000149	−3.05
P04066	FUCA1	$6.98x10^{-8}$	−2.99
A6QLB3	ITGA2B	0.00116	−2.41
A0A087WYV6	TSPAN6	0.000767	−2.33
A0A087WXT3	ZNF33B	0.043	−2.2
Q5SZC9	ABRACL	0.00212	−2.01
Q9H9K5	ERVMER34-1	0.00000389	−1.99
P07954-2	FH	0.0324	−1.98
E9PF78	ADAM22	$2.34x10^{-11}$	−1.86
P17174	GOT1	$4.04x10^{-8}$	−1.84
A0A3Q1LMU4	INHBB	0.0485	−1.79
E1BNP7	TFAP2D	0.0455	−1.74
H7BZJ3	PDIA3	0.0156	−1.69
A0A3Q1N7H1	CYRIB	0.0124	−1.6
F1MWN1	CSE1L	0.000909	−1.46

F5H6E2	MYO1C	0.00519	−1.46
Q5E998	CTSV	0.000679	−1.44
Q12805-5	EFEMP1	0.0233	−1.41
O95025	SEMA3D	0.000326	−1.39
A0A087WTM1	ROBO1	0.0000191	−1.27
Q92598-2	HSPH1	0.046	−1.24
P02750	LRG1	0.0408	−1.23
Q9UKM7	MAN1B1	0.0387	−1.02
C9JFR7	CYCS	0.0119	−1
Q0VD53	VPS26A	0.0157	1.01
Q9NYU2-2	UGGT1	0.0201	1.13
Q08945	SSRP1	0.0395	1.35
O00443	PIK3C2A	0.0186	1.37
F1N6D5	EFTUD2	0.012	1.4
Q6B856	TUBB2B	0.0425	1.44
P68431	H3C1	0.0474	1.45
Q5E9X4	LRRC59	0.0173	1.61
O97593	SMC1A	0.05	1.62
C9J6P4	ZC3HAV1	0.0184	1.66
P24627	LTF	0.0386	1.67
P40926	MDH2	0.0172	1.83
A0A0B4J1W3	NAA15	0.000341	1.89
O43765	SGTA	0.0487	1.89
H0Y6Z7	PTPRF	0.00948	1.95
Q9H6X2-3	ANTXR1	0.0000258	1.98
A0A3Q1M315	LOC790886	0.0000318	2.05
M0R1D6	DNAJB1	0.0000154	2.06
G5E9R5	ACP1	0.0000576	2.24
H7C1M2	SON	0.0476	2.24
P01833	PIGR	0.0499	2.35
F1MBG0	CARM1	0.00819	2.58
H7C3P9	COPS3	0.00135	2.59
A0A3Q1LPG0	A0A3Q1LPG0	0.00569	2.79
Q9NX76	CMTM6	0.00102	2.82
F1N2K1	PCYOX1	0.000303	3.03
K7EK07	H3-3B	0.00000319	3.04
Q96GE4	CEP95	0.0194	3.36
P0DPA2	VSIG8	0.0476	3.83
A0A494C0G8	SCLT1	0.0195	3.98
F1N0X6	TLL1	0.000963	4.49
A0A286YEY1	IGHA1	0.000000156	4.93

Table S4. Differential enrichment of EV proteins of E2P4 Group compared to C. Protein identified in EVs released from estrogen plus progesterone-treated RL95-2 compared to control treated RL95-2 cells.

Uniport Accession	Gene Name	Adjusted *p* Value	Fold Change
Q5VSY0-2	GKAP1	9.18E-05	−5.16
Q5E9M9	RHOT2	0.0327	−3.66
E5RGX5	STMN2	0.00314	−3.15
A0A3Q1M688	NDST1	1.7E-09	−3.02
P14324-2	FDPS	0.0386	−2.92
O94985-2	CLSTN1	0.00216	−2.69
Q14393-5	GAS6	0.0235	−2.63
Q9H9K5	ERVMER34-1	5.35E-06	−2.42
Q10471	GALNT2	0.0104	−2.32
Q56K13	SF3B5	0.05	−2.24
O75509	TNFRSF21	0.000117	−2.22
K7ELL7	PRKCSH	0.0275	−2.15
A0A087X0S5	COL6A1	0.00459	−2.05
P01024	C3	0.0469	−1.89
P51858	HDGF	0.00243	−1.87
P13667	PDIA4	0.0232	−1.75
A0A3Q1MRC3	EFNA5	0.0138	−1.65
F1MM32	QSOX1	0.00365	−1.6
Q8N474	SFRP1	0.0252	−1.47
Q969H8	MYDGF	5.84E-06	−1.45
J3KMY5	NPC2	0.0227	−1.37
Q9UQ74-2	PSG8	0.0349	−1.18
Q13541	EIF4EBP1	0.00552	1.6
Q3SZF2	ARF4	0.026	1.98
P04066	FUCA1	0.000341	2.66
E9PS42	CSRP1	0.0156	2.92
O75223	GGCT	0.000052	3.46
P01040	CSTA	0.029	3.7
Q9BZJ8	GPR61	0.0459	3.7
P25311	AZGP1	0.0434	3.89
A0A3Q1MM65	RPL4	0.000453	7.45

Table S5. Differential enrichment of EV proteins of E2 Group compared to C. Protein identified in EVs released from estrogen treated RL95-2 compared to control treated RL95-2 cells.

Uniport Accession	Gene Name	Adjusted *p* Value	Fold Change
Q5VSY0-2	GKAP1	0.0000202	−5.16
Q2KIX7	Q2KIX7	0.00118	−4.27
F1MER7	HSPG2	0.042	−3.93
Q14574	DSC3	0.0313	−3.46
Q86SQ4-2	ADGRG6	0.000284	−3.16
A6QLB3	ITGA2B	0.000198	−3.1
P25205	MCM3	0.0000168	−3.01
E5RGX5	STMN2	0.00278	−2.95
F1N672	TM9SF2	0.0344	−2.94
A0A3Q1NFD1	CACNA2D1	0.00319	−2.8
Q3ZC83	SLC29A1.1	0.0417	−2.61
Q53GA4	PHLDA2	0.0432	−2.53

G3N1U4	SERPINA3-3	0.05	−2.52
Q9H9K5	ERVMER34-1	0.00000159	−2.39
P00750	PLAT	0.0105	−2.28
Q10471	GALNT2	0.00937	−2.16
E9PB61	ALYREF	0.0373	−2.15
Q58D84	FSTL1	0.0491	−1.91
A0A087WYV6	TSPAN6	0.0474	−1.91
P19858	LDHA	0.0067	−1.89
P13667	PDIA4	0.00346	−1.89
A0A0G2JP90	NOMO1	0.0441	−1.85
F1MGE9	HYPK	0.0282	−1.79
P01024	C3	0.043	−1.76
A0A3Q1MNL9	TGFBI	0.00755	−1.75
A0A3Q1M688	NDST1	0.00194	−1.74
E9PF78	ADAM22	0.00000159	−1.63
P17174	GOT1	0.00091	−1.43
Q99523	SORT1	0.0409	−1.25
K7ENJ4	ATP5F1A	0.00908	2.11
E9PS42	CSRP1	0.0429	2.4
P40926	MDH2	0.000109	2.99
P12273	PIP	0.0271	3.26
Q6UWP8	SBSN	0.0443	3.58
P25311	AZGP1	0.0372	3.65
A0A0B4J231	IGLL5	0.000862	3.68
P31025	LCN1	0.023	4.05
A0A286YEY1	IGHA1	0.000264	4.31
F1N0X6	TLL1	0.0135	4.32
A0A494C0G8	SCLT1	0.0221	4.64
A0A3Q1MM65	RPL4	0.0171	5.44

Table S6. Differential enrichment of EV proteins of P4 Group compared to C. Protein identified in EVs released from progesterone treated RL95-2 compared to control treated RL95-2 cells.

Uniport Accession	Gene Name	Adjusted *p* Value	Fold Change
F1N2K1	PCYOX1	0.0186	−3.98
Q9H6X2-3	ANTXR1	0.0311	−2.13
A0A3Q1M688	NDST1	0.032	−1.97
Q13162	PRDX4	0.0457	−1.18
A0A3Q1M4Y2	CFAP57	0.028	6.03

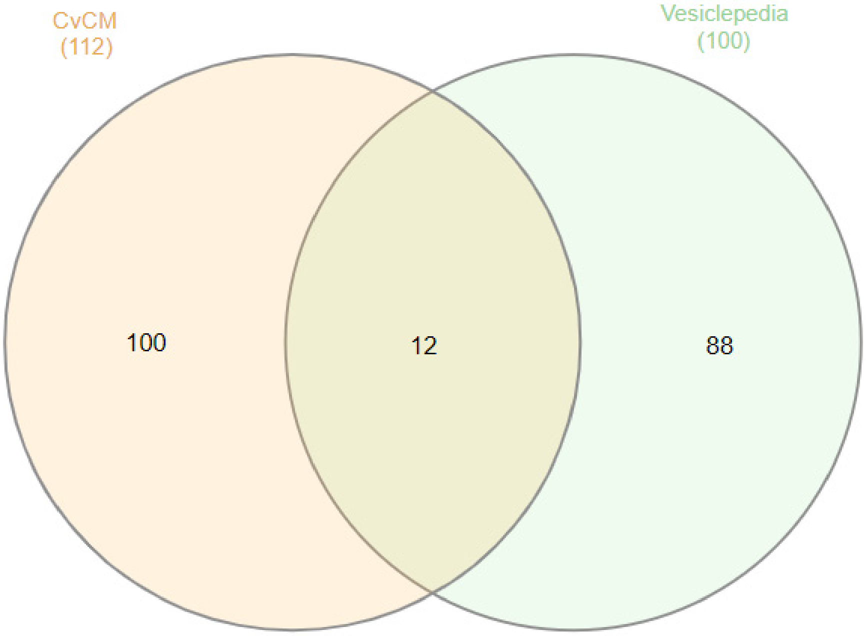

Figure S2. Ven diagram of proteins enriched in the Control EV group versus the conditioned media control compared to the top 100 Vesiclepedia EV markers [72].
